# Satellite evidence for changes in the NO_2_ weekly cycle over large cities

**DOI:** 10.1038/s41598-020-66891-0

**Published:** 2020-06-22

**Authors:** T. Stavrakou, J.-F. Müller, M. Bauwens, K. F. Boersma, J. van Geffen

**Affiliations:** 1 Royal Belgian Institute for Space Aeronomy, Avenue Circulaire 3, 1180 Brussels, Belgium; 2 Royal Netherlands Meteorological Institute, Satellite Observations, De Bilt, The Netherlands; 3 Wageningen University, Meteorology and Air Quality Group, Wageningen, The Netherlands

**Keywords:** Environmental chemistry, Chemistry

## Abstract

Anthropogenic activities, by far the largest source of NOx into the atmosphere, induce a weekly cycle of NO_2_ abundances in cities. Comprehensive analysis of the 2005–2017 OMI NO_2_ dataset reveals significant weekly cycles in 115 of the 274 cities considered. These results are corroborated by a full year of high-resolution TROPOMI NO_2_ observations. The OMI dataset permits us to identify trends in the weekly cycle resulting from NOx emissions changes. The data show a clear weakening of the weekly cycle over European and U.S. cities, an evolution attributed to the decline in anthropogenic emissions and the resulting growing importance of background NO_2_, whereas NO_2_ lifetime changes also play a minor role. In particular, the Sunday NO_2_ columns averaged over all U.S. cities are found to increase, relative to the weekly average, from 0.72 during 2005–2007 to 0.88 in 2015–2017. The opposite tendency is recorded in regions undergoing rapid emission growth. Multiyear simulations over the U.S. and the Middle East using the chemistry-transport model MAGRITTEv1.1 succeed in capturing the observed weekly cycles over the largest cities, as well as the observed long-term trends in the weekly cycle.

## Introduction

Nitrogen oxides (NOx = NO_2_ + NO) play a key role in atmospheric chemistry: they catalyse tropospheric ozone formation, they impact the self-cleaning capacity of the atmosphere, and they are precursors of secondary inorganic aerosol, with consequences for climate and human health^1^. Fossil fuel combustion is the dominant source of NOx in the atmosphere, estimated at ~60% of the global total, whereas emissions from vegetation fires, lightning and soils make up the rest^2^. Because of their relation to human activities, anthropogenic NOx emissions often display a weekly cycle, with reduced NOx levels in and around cities on rest days. Similar cycles have been also observed for other pollutants, e.g. aerosols^3,4^, and for meteorological parameters such as cloudiness^4,5^. The NOx weekly cycle was previously investigated using ground-based, aircraft and satellite measurements^6–8^, whereas spatial patterns observed from satellites were used to study the urban photochemistry with the help of models^9^. These studies, however, rely either on relatively short data records of at most several months^8,9^ or on satellite data from coarse resolution sounders^6,7^.

Here we use NO_2_ column data from two nadir-viewing satellite sensors, the Ozone Monitoring Instrument (OMI^10^) launched in July 2004, and the high-resolution Tropospheric Monitoring Instrument (TROPOMI), single payload of the Sentinel-5 Precursor (S5P) launched in October 2017^11^. Both sensors have an equatorial crossing time of ca. 13:40 (local time), and provide daily global coverage at resolutions of 13 × 24 km^2^ and 7.2 × 3.5 km^2^ (at nadir) for OMI and TROPOMI, respectively. The long data record compiled for this study (2005–2019) and the high resolution of both instruments allows to provide more robust information on the NO_2_ weekly cycle over a larger number of cities around the world, and to identify potential changes in these profiles throughout the years. The observed cycles are further evaluated using multiyear simulations of the MAGRITTE model^12^ over major cities in the U.S. and the Middle East.

## Results and Discussion

### Weekly cycles detected from OMI and TROPOMI

**Figure 1:**
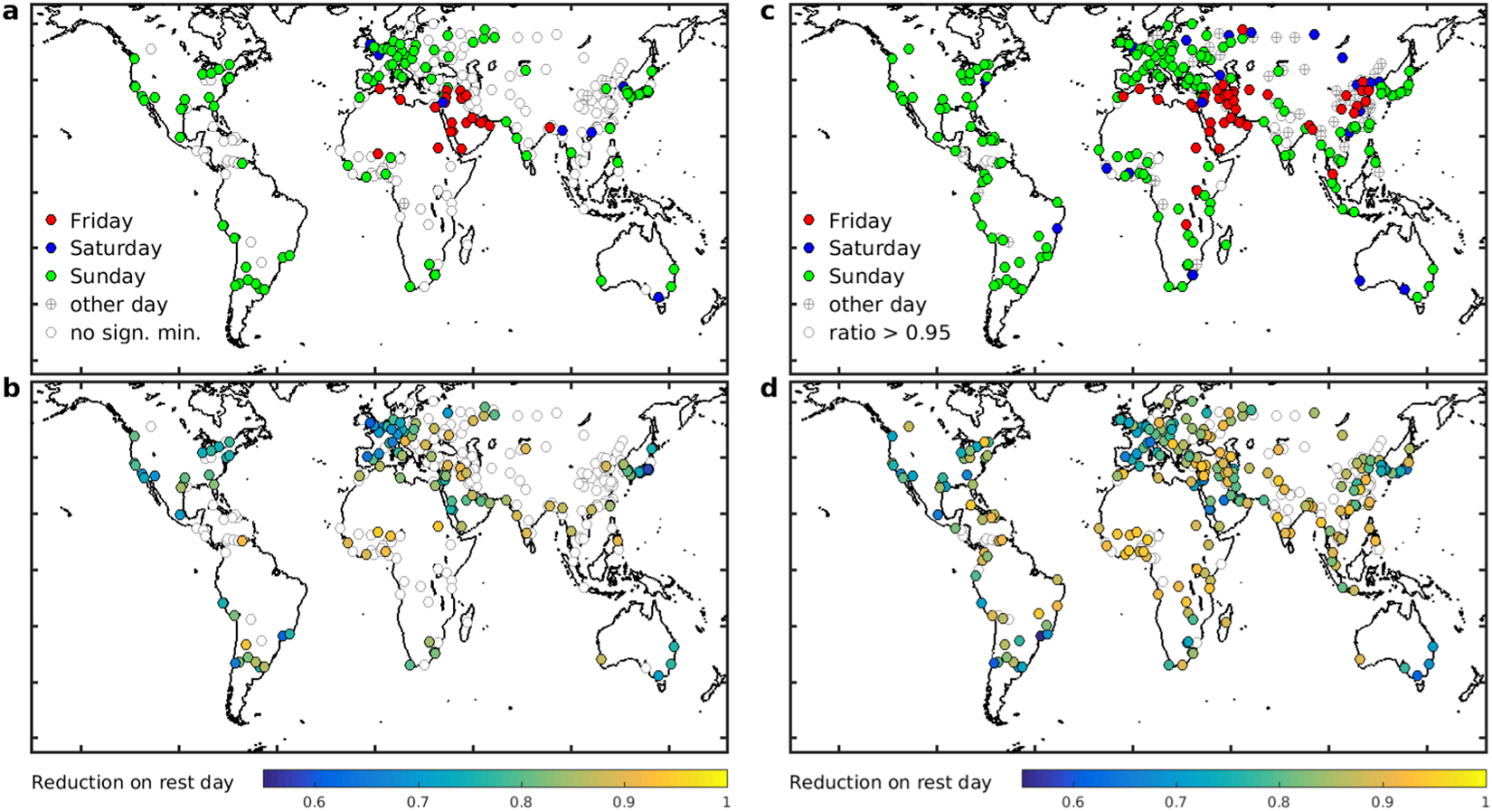
(**a**) Location of studied cities. The color indicates the day of minimum according to OMI NO_2_ data over 2005–2017. Open circles indicate cities for which the OMI minimum is not significant (see text). (**b**) Normalised NO_2_ column on the day of the minimum of OMI data. (**c**,**d**) Same as a and b for TROPOMI data over May 2018-April 2019. The numerical values of OMI and TROPOMI normalised columns are given in Supplementary Table S1. The map is generated using the shapefile landareas.shp from the mapping toolbox of MATLAB version R2013a (8.1.0.604), Natick, Massachussetts, The MathWorks Inc.

Based on the long-term OMI dataset, the NO_2_ weekly cycle at 115 of the 274 cities considered present a significant minimum (at the 99.7% confidence level), in the sense defined in Methods (Fig. 1a and Supplementary Table S1). In 112 among those 115 cities, the minimum occurs on a traditional rest day, the most frequent occurrences being Sunday (84), Friday (20), and Saturday (8). Minima on other days are very few (3) and not very pronounced (~10% below the weekly average).

**Figure 2:**
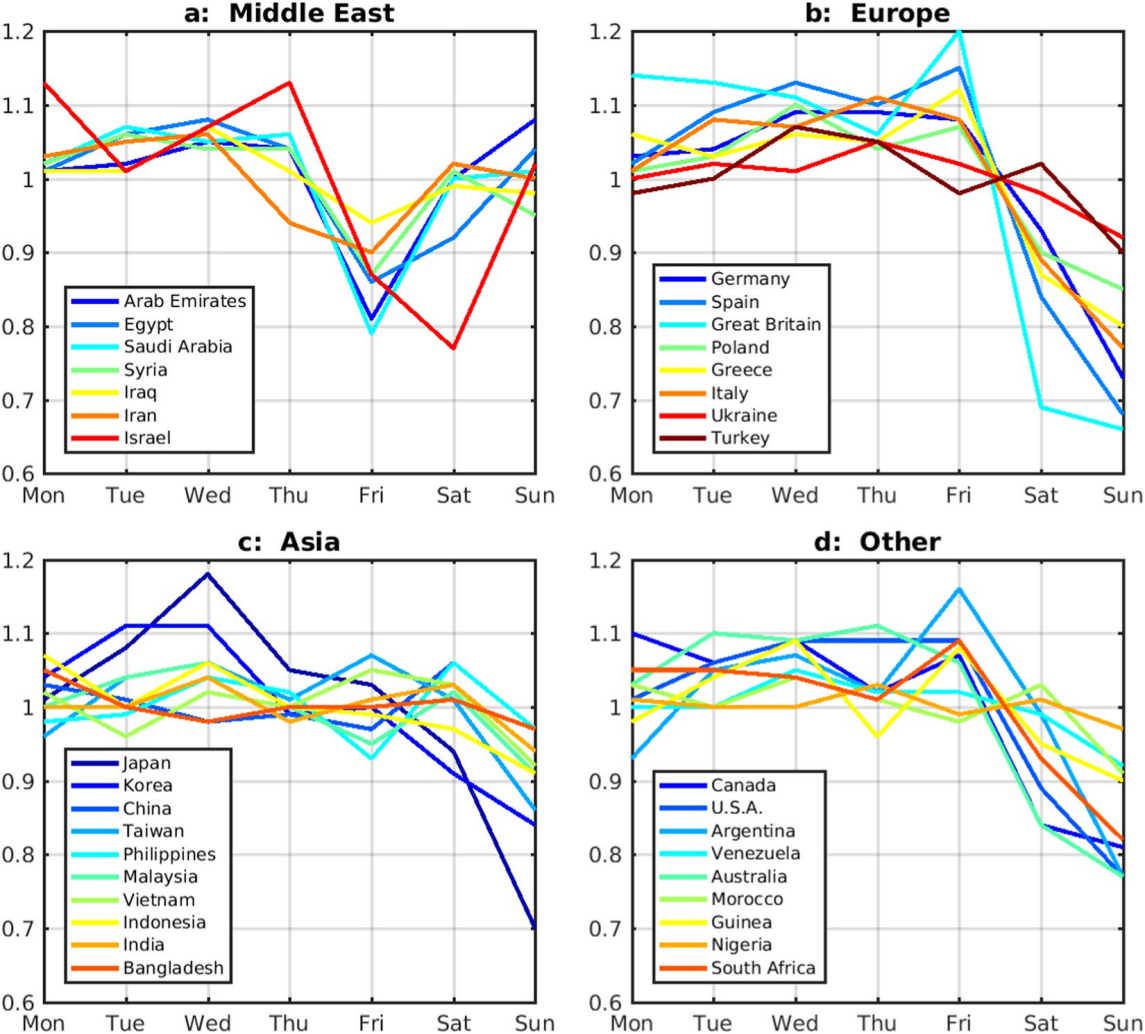
Mean normalised OMI NO_2_ weekly profiles averaged over all studied cities in countries of (**a**) the Middle East, (**b**) Europe, (**c**) Asia, and (**d**) countries in the Americas, Africa and Australia (cf. Supplementary Table S3).

As expected, and in agreement with previous satellite-based evaluations, large column decreases on Saturday and especially on Sunday are prevalent in cities of North America, Europe, Australia, Korea and Japan. In addition, the OMI dataset reveals that Sunday minima are also unambiguously common throughout Southern America and South Africa, and are even detected at cities in South Asia and Africa. This includes several predominantly Muslim countries adopting Monday-Friday as their regular workweek, such as Marocco, Turkey, Lebanon, and Pakistan (en.wikipedia.org/wiki/Workweek_and_weekend). To very few exceptions, the Sunday minimum goes hand-in-hand with lower-than-average NO_2_ columns on Saturday (Supplementary Table S1). The most pronounced minima are found in the megacities of Sao Paulo (Sunday: weekly average = 0.62), Tokyo (0.65), Los Angeles (0.68) and London (0.68), as well as in many other, mostly European, cities (Supplementary Table S2). In constrast, and despite showing very large NO_2_ columns, Chinese cities do not present a significant minimum. When averaging the weekly cycle over all large Chinese cities, the normalised column on Sunday is slightly below the average (0.97 ± 0.02, with 0.02 being the standard error). India and Indonesia display clear minima on Sunday (0.94 ± 0.02 and 0.91 ± 0.05, Fig. 2 and Supplementary Table S3).

In most predominantly Muslim countries (Middle East, North Africa and Bangladesh), a significant column decline is found on Friday, leading to normalised columns frequently ranging between 0.8 and 0.9, with the strongest decline (0.75) found for Mecca. The pronounced minimum detected on Saturday (0.77) in Jerusalem is similar to the ratio based on GOME NO_2_ columns reported by^6^, but is weaker than the reduction in Israeli cities found in^13^. This minimum is accompanied by a significant, but weaker reduction on Friday (ratio of 0.87), largely explained by the vicinity of predominantly Muslim city centers in Palestine and Jordan, in good agreement with^13^.

**Figure 3:**
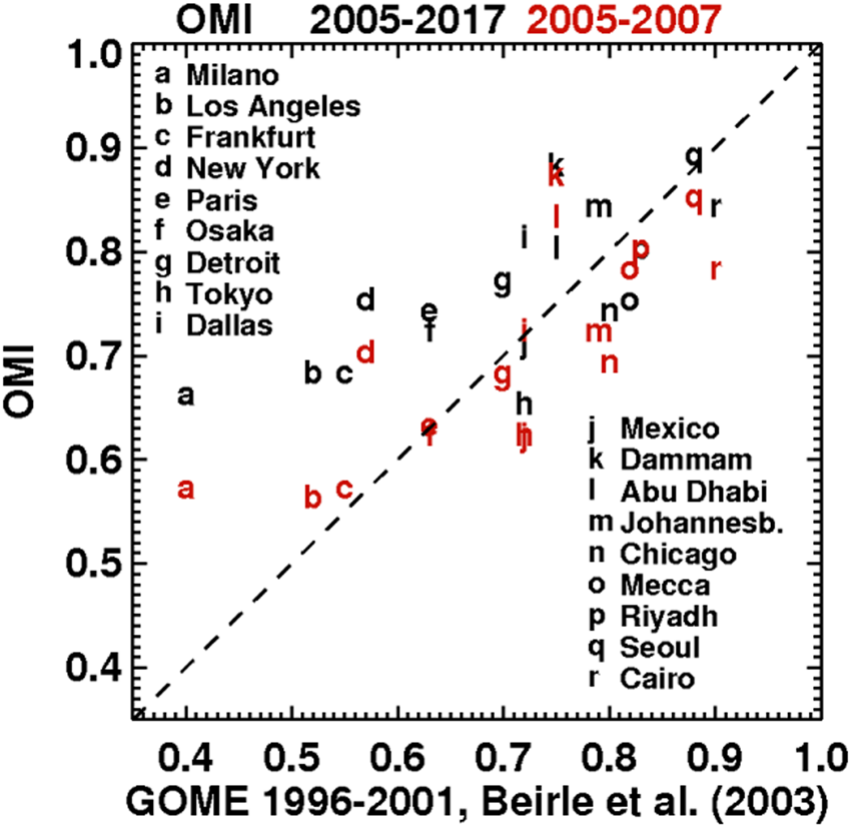
Column reduction during the rest day (Sunday or Friday) over 18 large cities reported in^6^ based on GOME 1996–2001 data, and based on OMI data from either 2005–2017 (black) or 2005–2007 (red).

As seen in Fig. 3, the OMI-based ratios are broadly in agreement with corresponding values based on GOME NO_2_ columns^6^, in spite of the lower resolution, different overpass time and different time period (1997–2001) of GOME data. The larger GOME pixels tend to dilute the anthropogenic signal and therefore weaken the NO_2_ column reduction on rest days. Higher GOME-based ratios are therefore expected based on this effect of resolution. Figure 3, however, shows that OMI-based ratios for the 2005–2017 period are on average 15% higher than the GOME-based values for most polluted cities (cities a-l on Fig. 3), and this difference even reaches 30–65% for the worst cases (cities a–d), in particular Milan. To a large extent, this discrepancy can be explained by the well-documented decline in NOx emissions and NO_2_ columns between the GOME and OMI time periods over those cities^14^. Indeed, when considering only OMI data from 2005–2007 (red symbols on Fig. 3), thereby mitigating this difference in time and therefore in NO_2_ levels, the agreement between the OMI- and GOME-based ratios is greatly improved, the correlation coefficient increasing from 0.53 to 0.62 and the mean bias being reduced from 19 to 7% for those very polluted cities. As discussed below, the weekend effect is favoured by higher anthropogenic emissions, which explains the weakening of the weekly cycle over the years over North America, Europe and Japan. The effect of resolution is nonetheless significant, likely explaining the generally higher GOME-based ratios for many cities, in comparison with OMI 2005–2017 (cities g-r on Fig. 3).

Over Los Angeles, the OMI-based ratio of 0.68 lies between previously reported values^8^ based on ground-based (0.82) and aircraft data (0.31). The OMI results for U.S. cities are consistent with previously reported values based on SCIAMACHY^7^ (Supplementary Table S2), with an average ratio for 7 cities of 0.68 for OMI over 2005–2007 (0.75 over 2005–2017) vs. 0.62 in the case of SCIAMACHY over 2003–2005.

The weekly cycles based on TROPOMI data agree reasonably well with those from OMI, as seen in Fig. 1 and Supplementary Table S1. Among the 332 cities for which TROPOMI time series are available, 191, 20 and 54 cities show a minimum (required to be at least 5% below the weekly mean) on Sunday, Saturday and Friday, respectively. Among the remainder, 56 cities show a minimum on another weekday, and 11 cities do not show a clear minimum. Since the TROPOMI dataset covers only one year, meteorology-induced natural NO_2_ variability has a stronger impact on the mean weekly cycle than for the multiyear OMI dataset. This explains the existence of important deviations to the expected weekly cycle at a large number of cities, e.g. in Russia, Latin America and China (Fig. 1c). The mean Sunday-to-week ratios from OMI (2005–2017) are very similar to those from TROPOMI over Europe (0.76 for OMI vs. 0.78 for TROPOMI on average for 21 cities, Supplementary Table S1) and North America (0.77 for both instruments, 18 cities). For cities having Friday as day of minimum, a good agreement is found between the two sensors (0.83 vs. 0.82).

### Weekly cycles and chemical lifetimes over North America

**Figure 4:**
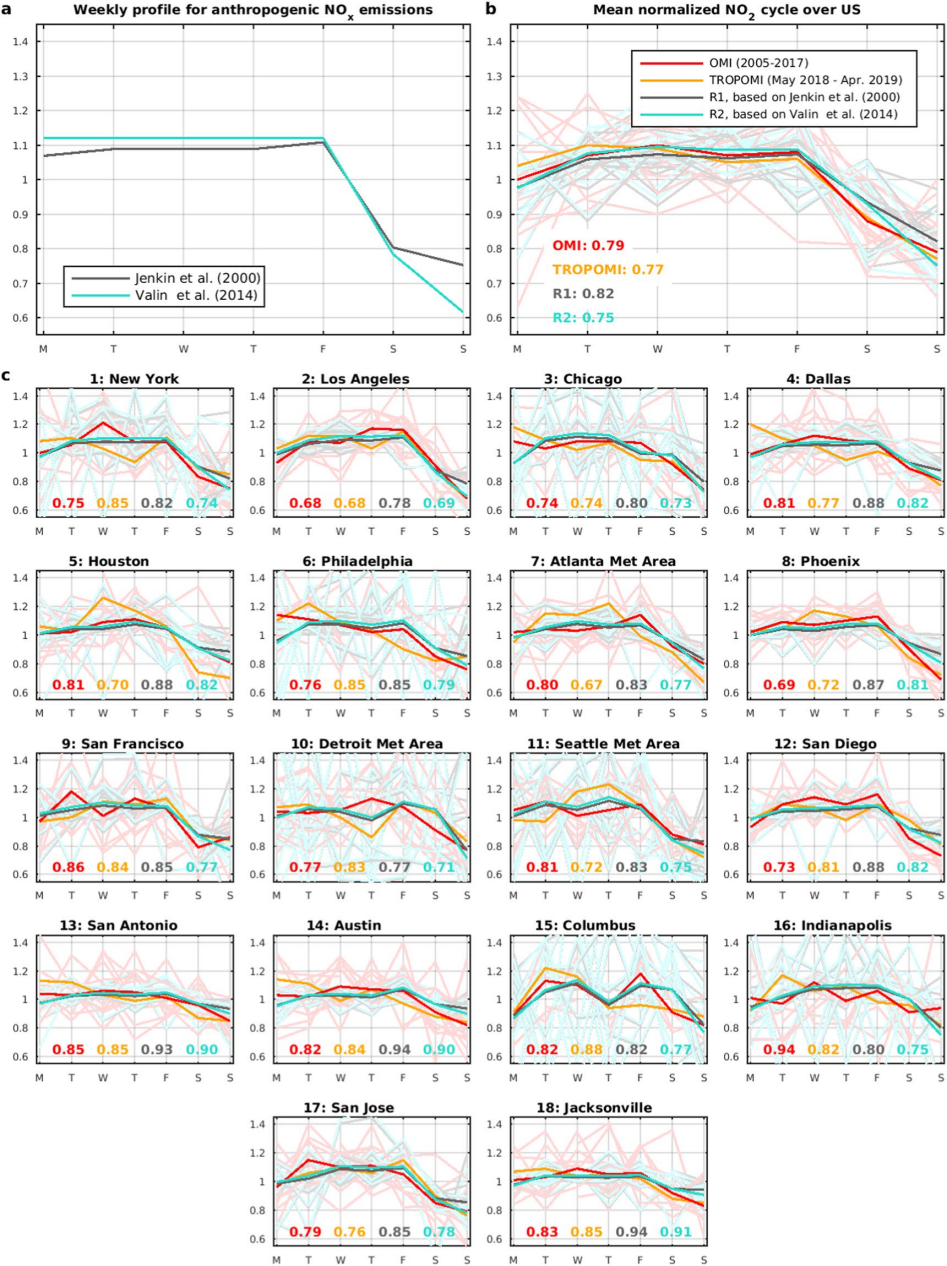
(**a**) Normalised weekly profiles of anthropogenic NOx emissions over the U.S. used in simulations R1 and R2. (**b**) Mean normalised weekly NO_2_ cycle for all U.S. cities (weighted by population) from OMI (2005–2017, red) and TROPOMI (orange), and from model runs R1 (black) and R2 (light blue). Dark red line: average OMI cycle for 2005–2017; pale lines: individual years. The normalised column on Sunday is given inset. (**c**) Same as b for individual cities ranked according to the population of their urban areas (en.wikipedia.org/wiki/List_of_United_States_urban_areas).

The modelled weekly cycles at U.S. cities are compared with observations on Fig. 4 for runs R1 and R2 (cf. Methods). The cities locations are displayed on Supplementary Fig. S1. Overall, the model reproduces well the observed weekly cycles across the different cities. The mean observed Sunday-to-week ratio for OMI (0.79) lies between the modelled values of the R1 (0.82) and R2 (0.75) simulations. The modelled ratios on Saturday (0.93) overestimate the observed value (0.88), suggesting that a stronger emission reduction could have been used in the model. The interannual variability of OMI weekly profiles is high, especially at the northernmost cities (>36°N) where higher solar zenith angles and cloudiness lead to larger retrieval uncertainties and stronger natural variability. In addition, the lower photolysis frequencies and generally stronger winds at those latitudes enhance the role of atmospheric transport and therefore the mixing of imported air with local emissions. It is therefore not surprising that the 3 cities with the lowest interannual variability (standard deviation of ~0.1), Los Angeles, Phoenix and San Diego, show also the most pronounced weekly cycle (Sunday ratio ~0.7).

As seen in Fig. 4, the performance of model run R2 is excellent at the largest agglomerations (cities 1–7 on Fig. 4), with a mean absolute difference (MAD) on Sunday of 0.016, whereas run R1 systematically overestimates the ratio (MAD = 0.07). At the other, smaller cities, R2 overestimates the OMI ratio at cities with low interannual variability (mean bias ~0.08 for 5 cities), and underestimates it when variability is high (bias = −0.1, 5 cities). Note that similar biases are found with respect to TROPOMI for these two groups of cities (biases 0.05 and −0.07).

**Figure 5:**
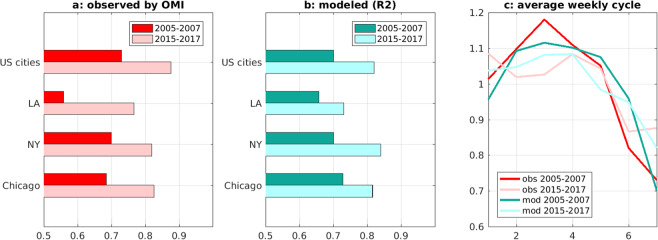
(**a**) Sunday-to-week column ratios averaged over 2005–2007 and over 2015–2017 for U.S. cities (average of 18 cities weighted by population), and for New York, Los Angeles and Chicago as observed by OMI. (**b**) Same for ratios calculated with the MAGRITTE model simulation R2. (**c**) Average observed and modelled weekly cycle for U.S. cities over 2005–2007 and 2015–2017.

The NOx emission decline over 2005–2017 has led to a significant weakening of the weekly cycle, as shown by the comparison of Sunday-to-week column ratios for the periods 2005–2007 and 2015–2017 (Fig. 5). On average for all cities, the ratio has increased by 22% (from 0.72 to 0.88), in excellent agreement with the model prediction (+20%). This result reflects the non-linear relationship between anthropogenic NOx emissions and NO_2_ columns, due to (1) the contribution of natural emissions (either local or imported) to the NO_2_ column above cities, and (2) chemical feedbacks of the NOx-OH system. Although the contribution of natural sources to total emissions is very small locally (less than ~1% for most cities even when using a 40 km radius), the contribution of non-local emissions to NO_2_ columns above cities is not negligible, especially when considering the increasing vertical sensitivity of OMI NO_2_ with altitude. Indeed, a significant fraction of the retrieved column lies in the middle and upper troposphere^15^, where long-range transport of NO_2_ is more effective, bringing rural air above urban areas.

Since natural emissions have no weekly cycle, the decline of anthropogenic emissions implies a dampening of the NO_2_ weekly cycle. In addition, the emission decrease generally leads to lower OH levels (primarily through the NO + HO_2_ reaction) and therefore to longer NOx lifetimes^16^, increasing the slope (*β*) of the relationship between an anthropogenic NOx emission change (Δ*E*) and the resulting NO_2_ column change (ΔΩ),

1 $$\frac{\Delta E}{E}=\beta \cdot \frac{\Delta \Omega }{\Omega }.$$

Based on calculations by^17^ using a regional model, *β* is estimated to increase by a factor of ~2.2 when decreasing the anthropogenic emission from ~160 · 10^10^ molec.cm^−2^s^−1^ to 20 · 10^10^ molec.cm^−2^s^−1^; this translates into a 22% increase in *β* for the estimated emission decrease between 2005–2007 and 2015–2017 (factor of 1.7 according to US EPA^18^, in line with recent assessments^17,19,20^). This change in *β* is in excellent agreement with our model results (+20%). Only a small part of this change is due to chemical feedbacks, as pointed out by^17^. Using MAGRITTE model simulations for the years 2006 and 2016 (with identical meteorological fields and natural emissions for the two years in order to isolate the anthropogenic impact), we calculate that chemical feedbacks can increase *β* by only about 4–8% at the largest U.S. cities (Supplementary Fig. S2). The rest of the change in *β* (>~10%) is therefore due to other factors, namely the increased contribution of the background to the tropospheric columns.

### Middle East: observed and simulated weekly cycles

**Figure 6:**
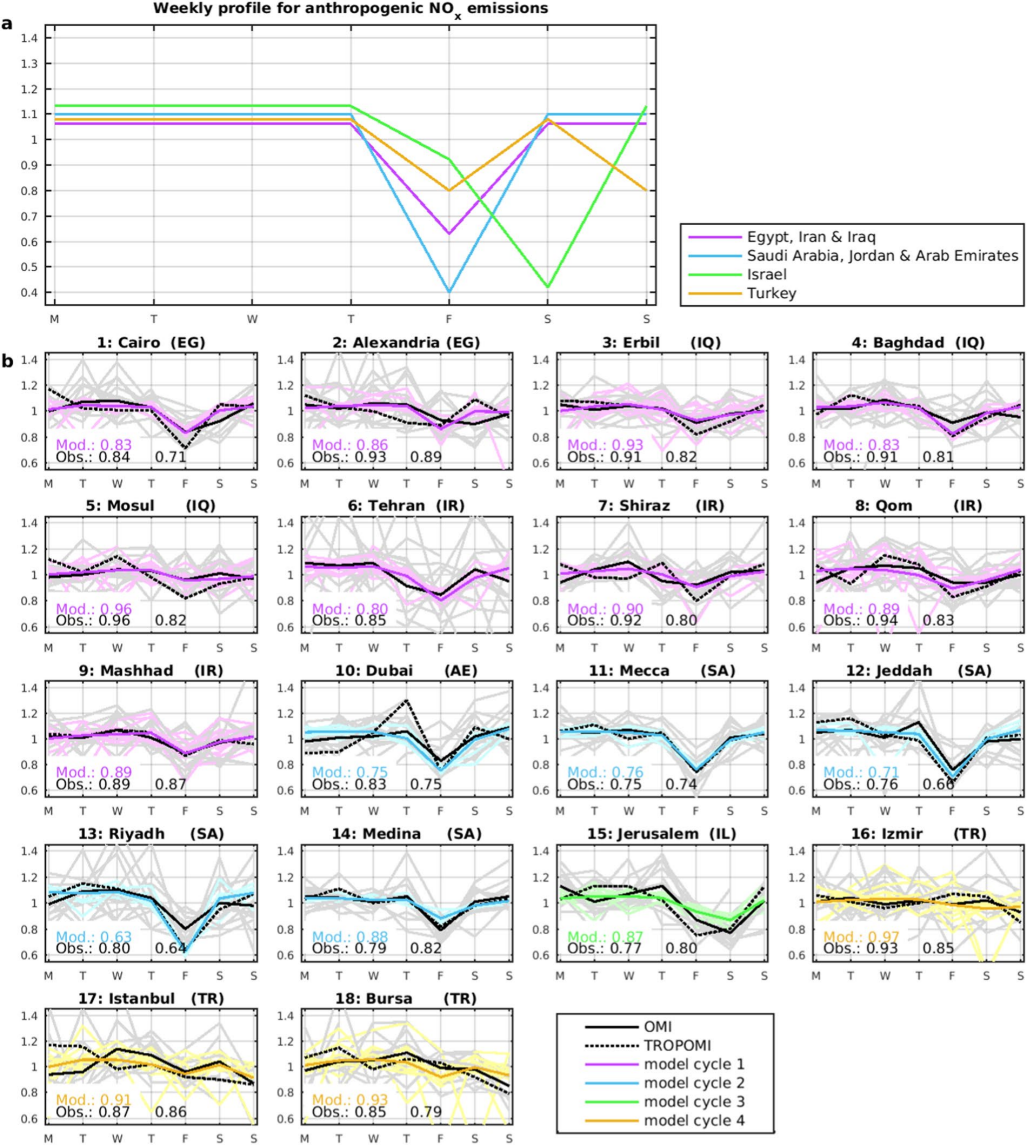
(**a**) Normalised weekly profiles of anthropogenic NOx emissions for Middle East countries used in the simulations (see main text). (**b**) Mean normalised weekly NO_2_ cycle in Middle East cities observed by OMI (black), TROPOMI (dashed black) and predicted by the model (coloured lines). The model uses distinct weekly emission profiles for Egypt, Iran and Iraq (magenta), Arab Emirates, Jordan, and Saudi Arabia (blue), Israel (green), and Turkey (yellow). Thick black line: average OMI 2005–2017; thin grey lines: individual years. The day-of-minimum normalised column is given inset for the model, OMI, and TROPOMI.

The NO_2_ weekly cycles at 18 Middle Eastern cities are compared with the model on Fig. 6. A fair agreement is found between the OMI-based climatology and TROPOMI at most cities, considering the large interannual variability. Stronger Friday minima are however detected by TROPOMI over Iraqi cities, Erbil (0.82 vs.0.91), Baghdad (0.81 vs. 0.91), and Mosul (0.82 vs. 0.96). At those cities, however, TROPOMI results are very consistent with OMI results from year 2017, with Friday ratios of 0.84, 0.84 and 0.81, respectively. These results suggest a strengthening of the weekly cycle since 2005, likely due to the increase in NOx levels (caused by increasing emissions) and the resulting declining background contribution and NO_2_ lifetime shortening over Iraqi cities, following the lines discussed above (Eq. 1). The NO_2_ columns have increased by factors of 1.6 in Baghdad and 2.3 in Erbil between 2005 and 2017. Comparatively much lower NO_2_ trends were observed over Saudi Arabia, Israel and Turkey, where the normalised weekly NO_2_ cycle remained stable.

Over Turkey, the weekly cycle is relatively weak, with a primary minimum on Sunday (0.89 on average for all cities) and a secondary one on Friday (0.98). Beirut (Lebanon, Supplementary Table S1) shows also a minimum on Sunday (0.8) but not on Friday. Jerusalem shows a pronounced Saturday minimum (0.77), and also low normalised columns on Friday (0.84).

The model succeeds reasonably well in capturing the observed cycles, in particular at the megacities Cairo, Tehran and Istanbul. The pronounced emission minimum assumed on Friday for Saudi Arabia and the Emirates (ratio of 0.41) leads to an excellent agreement at Mecca but to a too strong reduction for Saudi cities (ratio of 0.74 vs. 0.79 on average) as well as for the Emirates (0.78 vs. 0.81). The large difference between the emission reduction and the column reduction on Friday indicates a large value of *β* in the model (Eq. 1), possibly overestimated at the smaller cities (e.g. Medina and Mecca) occupying only a small fraction of a model gridcell. Therefore, although the adopted weekly cycle of emissions for these two countries (also Israel) leads to a fair agreement with the data, a fine resolution model would be better suited for those cities.

## Conclusion

NOx emission trends cause significant changes in the weekly cycle, which are detected in the satellite data. The well-documented decline of NO_2_ levels over many industrialised countries leads to an increased contribution of the background to NO_2_ columns. It also often leads to lower OH levels and hence longer NO_2_ lifetimes, which further change the relationship between NOx emissions and NO_2_ levels. These effects explain the weakening of the NO_2_ weekly cycle over Europe and the U.S. over the OMI measurement period, as well as a great deal of the differences between the GOME-, OMI- and TROPOMI-based weekly cycles. They also account for the strenghtening of the cycle over Iraqi cities over the years, in response to the fast emission increase. The fairly good simulation of these effects for U.S. cities during 2005–2017 suggests an adequate model representation of the relevant processes, even though model limitations such as the likely too coarse model resolution are acknowledged. We therefore recommend the inclusion of weekly cycles of emissions as a standard procedure for chemistry-transport models, in order to improve the model performance regarding temporal variability. More work will be needed to refine the model representation of the weekly cycle in models, e.g. through detailed model analyses in other parts of the world. A finer-resolution model might be preferred in future studies, especially when several years of TROPOMI data will become available, enabling temporal averaging and the derivation of weekly cycles for smaller cities.

## Methods

### Satellite NO_2_ observations and data processing

The OMI Quality Assurance for Essential Climate Variables (QA4ECV) NO_2_ dataset is based on revised spectral fitting features accounting for improved absorption cross sections, instrument calibration, and surface effects^21–23^. The OMI columns are averaged for each day of the week and each year over 2005–2017 for every city of more than 7 · 10^5^ inhabitants (3 · 10^6^ for China and India) according to the GeoNames database (www.geonames.org), using data lying within 40 km of the city. The data are processed according to the data quality recommendations^24^. We excluded annual averages built on five (or less) valid individual measurements, or when the relative uncertainty exceeds 120% of the annual average. For each city and year, the weekly cycle is normalised to the weekly mean. Those normalised weekly cycles are used to calculate a 2005–2017 climatology, requiring that at least two years of data are available for each day of the week. This climatological cycle presents a minimum which is considered significant when it differs from the mean (equal to 1) by more than 3*σ*, where *σ* is the standard error. Hereafter, the weekly cycles presenting such a significant minimum will be qualified as significant.

The TROPOMI NO_2_ product benefits from the developments of the OMI QA4ECV retrieval^25^. We use TROPOMI data version 1.2.2 and 1.3.0, publicly available from https://s5phub.copernicus.eu. Only data with a quality flag value higher than 0.75 are retained. The first complete year of TROPOMI data (May 2018-April 2019) is processed similarly as for OMI.

### Simulations of the NO_2_ weekly cycle with the MAGRITTE model

Regional simulations of tropospheric composition over the U.S. and the Middle East are conducted with the Model of Atmospheric composition at Global and Regional scales using Inversion Techniques for Trace gas Emissions (MAGRITTEv1.1) for 2005–2017 at a resolution of 0.5° × 0.5° with 40 vertical levels. MAGRITTE uses lateral boundary conditions from the IMAGES model^26–29^, and inherits most model parameterisations from this model. Chemistry is solved by a 4^*th*^-order Rosenbrock solver of the Kinetic PreProcessor^30^. The model chemical mechanism is described in ref. ^12^. The model uses ECMWF ERA-Interim meteorological fields^31^. Anthropogenic NOx emissions are obtained from the HTAPv2 2010 inventory^32^ at 0.1° regridded at the model resolution. Over the U.S., the emissions are scaled to the annual emissions reported by the EPA National Emissions Inventory^18^.

Two simulations are performed for the U.S., using different weekly cycles of anthropogenic NOx emissions. According to the profile of ref. ^33^ adopted in simulation R1, which is based on real data for the main emission categories (e.g., fuel consumption) and a small set of default profiles for minor sources, the normalised emissions on Saturday and Sunday amount to 0.8 and 0.75 relative to the weekly mean, respectively. A stronger emission decrease was adopted in the profile of ref. ^9^ of simulation R2, with a normalised emission of 0.7 for the week-end. We adopted normalised emissions of 0.784 on Saturday and 0.616 on Sunday in run R2, in order to reflect the expected lower Sunday emission (Fig. S1). For the Middle East, relevant information on the weekly cycle is unavailable. We adopted country-dependent NOx emission profiles, with strong emission minima (0.41) on Friday in Saudi Arabia and United Arab Emirates, and on Saturday in Israel, and more moderate minima (0.63) on Friday in most other Muslim countries. In Turkey, we applied similar emission reduction on Friday and Sunday (0.8, Fig. S1). These profiles have been loosely adjusted to match the observed weekly cycles for the different countries.

The simulated monthly averaged NO_2_ columns at the local OMI overpass time (13:30) are calculated from daily values by accounting for the averaging kernels and sampling times of valid OMI data. Those gridded columns are then mapped onto the areas used for the satellite data processing (40 km radius around the city centers) and compared.

## Supplementary information

Supplementary Information.

## Data Availability

The QA4ECV dataset from OMI (Version 1.1) is publicly available at 10.21944/qa4ecv-no2-omi-v1.1. This publication contains modified Copernicus Sentinel data 2018–2019. TROPOMI data version 1.2.2 and 1.3.0 used is available at https://s5phub.copernicus.eu.
