# Supplementary material for: Satellite evidence for changes in the NO2 weekly cycle over large cities
Source: Sci Rep. 2020 Jun 22;10:10066. doi: 10.1038/s41598-020-66891-0 (PMC7308373; doi:10.1038/s41598-020-66891-0)
Supplement: Supplementary file 1 — Supplementary Information. [file 41598_2020_66891_MOESM1_ESM.pdf]

Satellite evidence for changes in the NO_2 weekly cycle over large cities

T. Stavrakou[1⋆], J.-F. Müller[1], M. Bauwens[1], K. F. Boersma[2,3], J. van Geffen[2]

[1] Royal Belgian Institute for Space Aeronomy, Avenue Circulaire 3, 1180, Brussels, Belgium

[2] Royal Netherlands Meteorological Institute, Satellite Observations, De Bilt, the Netherlands

[3] Wageningen University, Meteorology and Air Quality Group, Wageningen, the Netherlands

⋆ E-mail : Trissevgeni.Stavrakou@aeronomie.be

Contents

1. Normalised weekly ratios per city (Table S1)
2. Mean normalised NO_2 weekly profiles per country (Table S2)
3. Comparisons to previous studies (Table S3)
4. Locations of studied cities in U.S. and Middle East (Figure S1)
5. Calculated ratio of Sunday NOx lifetime to the average NOx lifetime in 2006 and 2016 (Figure S2)

Table S1: Ratios of the observed NO_2 column on Friday, Saturday and Sunday to the weekly average for all cities with a significant minimum in OMI data (colored zone). The country codes are also provided (second column).

		OMI			TROPOMI		
		**Fri**	**Sat**	**Sun**	**Fri**	**Sat**	**Sun**
Abu Dhabi	AE	0.80	0.98	1.07	0.81	1.03	1.04
Aleppo	SY	0.87	1.02	0.96	0.95	1.03	1.08
Algiers	DZ	0.86	1.00	1.02	0.76	1.00	1.05
Amman	JO	0.76	0.93	1.07	0.67	1.00	1.13
Cairo	EG	0.84	0.93	1.06	0.71	1.05	1.03
Damascus	SY	0.83	0.99	0.94	0.93	1.01	1.04
Dammam	SA	0.88	1.01	1.09	0.81	0.90	1.01
Dubai	AE	0.83	1.02	1.09	0.75	1.09	1.00
Erbil	IQ	0.91	0.98	1.00	0.82	0.92	1.05
Jeddah	SA	0.76	0.98	1.00	0.66	1.01	1.03
Kahriz	IR	0.88	1.06	1.03	0.90	0.91	0.96
Khartoum	SD	0.94	0.99	1.01	0.88	0.95	1.02
Khulna	BD	0.89	1.05	1.03	1.03	0.97	0.94
Mecca	SA	0.75	1.01	1.04	0.74	0.99	1.05
Medina	GH	0.79	1.01	1.05	0.82	0.98	1.03
Muscat	OM	0.84	0.99	1.05	0.89	0.93	1.02
Riyadh	SA	0.80	1.00	0.98	0.64	0.95	1.07
Sanaa	YE	0.86	0.95	1.01	0.87	0.97	1.06
Tripoli	LY	0.83	0.99	1.04	0.89	0.96	1.10
Birmingham	GB	1.08	0.61	0.63	1.00	0.96	0.72
Jerusalem	IL	0.87	0.77	1.02	0.75	0.80	1.13
Mandalay	MM	1.02	0.87	1.01	1.03	1.03	1.04
Paris	AU	1.04	0.70	0.74	1.25	0.79	0.72
San Francisco	US	1.06	0.79	0.86	1.13	0.87	0.84
Seoul	KR	0.99	0.86	0.88	1.22	1.04	0.72
Zhongshan	CN	0.97	0.88	1.01	0.97	0.93	1.00
Abidjan	CI	1.08	0.96	0.89	0.99	1.07	0.95
Almaty	KZ	1.04	0.99	0.90	1.08	1.00	0.94
Arequipa	PE	1.06	1.07	0.82	1.01	1.08	0.90
Atlanta	US	1.14	0.92	0.80	0.99	0.88	0.67
Austin	US	1.06	0.91	0.82	0.97	0.88	0.84
Bangkok	TH	0.99	1.04	0.87	1.07	0.99	0.83
Barcelona	ES	1.17	0.89	0.68	1.12	0.91	0.70
Bengaluru	IN	0.97	1.00	0.90	1.09	0.99	0.90
Benin City	NG	1.04	1.01	0.92	1.01	1.03	0.94
Berlin	DE	1.09	0.94	0.73	1.03	0.93	0.91
Brisbane	AU	1.05	0.89	0.76	1.05	0.82	0.70
Brussels	BE	1.03	0.76	0.65	1.16	0.83	0.74
Bucharest	RO	1.04	1.00	0.89	1.13	0.96	0.86
Budapest	HU	1.05	0.94	0.84	1.09	0.90	0.78
Buenos Aires	AR	1.18	0.99	0.76	1.15	0.88	0.69
Bursa	TR	0.99	0.98	0.85	1.03	0.92	0.79
Busan	KR	1.00	0.93	0.80	1.10	0.93	0.78
Casablanca	MA	0.99	1.03	0.86	0.97	1.02	0.84
Cebu City	PH	1.02	1.01	0.90	1.02	0.99	0.92
Chelyabinsk	RU	0.98	1.11	0.80	1.03	0.95	0.98
Chicago	US	1.07	0.92	0.74	0.95	0.94	0.74
Conakry	GN	1.08	0.95	0.90	1.08	0.97	0.95
Cordoba	ES	1.09	1.00	0.82	1.14	0.92	0.83
Daegu	KR	1.07	0.90	0.78	1.14	0.99	0.72
Dallas	US	1.06	0.89	0.81	1.01	0.93	0.77
Donetsk	RU	1.06	0.99	0.90	0.92	0.92	0.91
Durban	ZA	1.07	1.02	0.82	1.10	0.84	0.86
Frankfurt	DE	1.12	0.91	0.68	1.03	1.04	0.79
Gaziantep	TR	1.00	1.00	0.90	1.02	1.04	0.93
Hamburg	DE	1.03	0.90	0.79	1.05	1.14	0.83

Table S1: Cont'd

		OMI			TROPOMI		
		**Fri**	**Sat**	**Sun**	**Fri**	**Sat**	**Sun**
Jacksonville	US	1.06	0.92	0.83	1.02	0.88	0.85
Johannesburg	ZA	1.06	0.94	0.84	0.99	0.95	0.73
Kano	NG	0.97	1.02	0.93	0.95	0.99	0.99
Karachi	PK	0.98	1.08	0.85	1.03	1.21	0.79
Kiev	UA	1.01	0.96	0.88	1.05	0.95	0.84
Kitakyushu	JP	1.01	1.04	0.82	1.04	0.99	0.98
Koeln	DE	1.11	0.90	0.77	1.13	0.88	0.73
Krakow	PL	1.11	0.87	0.80	1.07	0.97	0.74
Lima	PE	0.97	1.03	0.77	1.07	0.97	0.71
London	GB	1.20	0.71	0.68	1.15	0.86	0.87
Los Angeles	US	1.16	0.91	0.68	1.15	0.87	0.68
Madrid	ES	1.18	0.78	0.64	1.16	0.92	0.66
Melbourn	CA	1.09	0.71	0.74	1.23	0.90	0.62
Mendoza	AR	1.01	1.01	0.82	1.08	0.99	0.80
Mexico City	MX	1.12	1.05	0.71	1.18	1.15	0.65
Milano	IT	1.14	0.71	0.66	1.17	0.90	0.64
Montevideo	UY	0.90	1.01	0.86	1.03	0.86	0.73
Montreal	CA	1.08	0.89	0.78	1.13	0.83	0.78
Moscow	RU	1.30	0.93	0.70	1.12	1.03	0.76
Mumbai	IN	1.01	1.04	0.89	1.00	1.02	0.97
Munich	DE	1.08	0.93	0.69	1.05	0.95	0.80
Nagoya	JP	1.04	0.92	0.68	1.07	0.96	0.74
Napoli	IT	1.03	0.99	0.85	1.07	0.93	0.80
New York	US	1.07	0.85	0.74	1.13	0.90	0.85
Perm	RU	1.05	1.02	0.81	0.85	0.89	0.97
Niamey	NE	0.95	1.02	0.95	1.05	1.00	0.94
Perth	AU	1.11	0.90	0.88	1.04	0.90	0.91
Philadelphia	US	1.04	0.85	0.76	0.90	0.82	0.85
Phoenix	US	1.13	0.90	0.69	1.06	0.84	0.72
Pietermaritzburg	ZA	1.02	0.85	0.81	0.98	0.77	0.87
Prague	CZ	1.06	0.93	0.73	1.13	0.87	0.84
Ras Bayrut	LB	1.12	0.97	0.80	1.00	1.12	0.88
Rio de Janeiro	BR	1.03	0.96	0.76	1.09	0.92	0.69
Rome	IT	1.04	0.97	0.78	1.11	0.97	0.75
Rosario	AR	1.07	1.05	0.86	1.15	0.90	0.86
Sakai	JP	1.05	0.87	0.72	1.01	0.87	0.66
Samara	RU	1.12	0.97	0.89	1.08	0.91	0.84
San Antonio	CL	1.01	0.96	0.85	1.02	0.87	0.85
San Diego	CR	1.16	0.85	0.73	1.09	0.98	0.81
San Jose	PH	1.05	0.85	0.79	1.15	0.90	0.76
San Miguel de Tucuma	AR	1.11	0.94	0.93	1.03	0.94	0.86
Santiago	CL	1.12	0.93	0.65	1.24	0.77	0.60
Sao Paulo	BR	1.12	0.98	0.62	1.15	0.79	0.52
Sapporo	JP	1.17	0.91	0.75	1.11	0.95	0.86
Seattle	US	1.09	0.88	0.81	1.08	0.84	0.72
Sydney	AU	1.03	0.84	0.77	1.09	0.86	0.69
Tainan	TW	1.05	0.97	0.84	1.06	0.97	0.88
Tokyo	JP	1.04	0.94	0.65	0.86	0.95	0.67
Toronto	CA	1.08	0.75	0.74	1.16	0.84	0.73
Turin	IT	1.09	0.86	0.78	1.07	0.93	0.72
Ufa	RU	1.07	0.92	0.88	0.89	1.00	0.85
Valencia	ES	1.02	1.13	0.90	1.00	0.91	0.75
Vienna	AT	1.10	0.91	0.77	1.12	0.85	0.82
Warsaw	PL	1.05	0.88	0.85	1.12	0.97	0.74
Zagreb - Centar	HR	1.01	0.97	0.91	1.01	1.01	0.82
Zibo	CN	0.94	0.97	0.89	0.86	1.10	1.03

Table S2: Ratio of the rest-day column to the weekly average for cities with the most pronounced OMI NO_2 weekly cycle. The ratios are calculated based on annually averaged OMI (2005-2017) and TROPOMI (May 2018-April 2019) columns. The ratio based on 2005-2007 OMI data is given within parentheses. Notes: [a] based on GOME 1996-2001 [Beirle et al., 2003]; [b] based on SCIAMACHY 2003-2005 [Kaynak et al., 2009]; [c] uses ground-based data for 2010 [Kim et al., 2016]; [d] based on aircraft data for 2010 [Kim et al., 2016].

City, Country	OMI	TROPOMI	previous estimates
Rest day: Sunday			
Sao Paulo, Brazil	0.62 (-)	0.52	
Birmingham, U.K.	0.63 (0.49)	0.72	
Madrid, Spain	0.64 (0.56)	0.66	
Brussels, Belgium	0.65 (0.53)	0.74	
Tokyo, Japan	0.65 (0.61)	0.76	0.72[a]
Santiago, Chile	0.65 (0.62)	0.60	
Milano, Italy	0.66 (0.57)	0.64	0.4[a]
Los Angeles, U.S.	0.68 (0.56)	0.68	0.52[a], 0.62[b], 0.82[c], 0.31[d]
Nagoya, Japan	0.68 (0.60)	0.74	
Frankfurt, Germany	0.68 (0.57)	0.79	0.55[a]
London, U.K.	0.68 (0.76)	0.87	
Barcelona, Spain	0.68 (0.62)	0.70	
Munich, Germany	0.69 (0.68)	0.80	
Phoenix, U.S.	0.69 (0.63)	0.72	0.50[b]
Moscow, Russia	0.70 (0.76)	0.76	
Mexico City, Mexico	0.71 (0.66)	0.65	0.72[a]
Osaka, Japan	0.73 (0.74)	0.65	0.63[a]
Prague, Czech Rep.	0.73 (0.73)	0.84	
Paris, France	0.74 (0.66)	0.72	0.63[a]
Chicago, U.S.	0.74 (0.68)	0.74	0.80[a], 0.60[b]
New York, U.S.	0.75 (0.70)	0.83	0.57[a], 0.70[b]
Rest day: Friday			
Mecca, Saudi Arabia	0.75 (0.78)	0.74	0.82[a]
Jeddah, Saudi Arabia	0.76 (0.70)	0.66	
Amman, Jordan	0.76 (0.77)	0.67	
Medina, Saudi Arabia	0.79 (0.80)	0.82	
Riyadh, Saudi Arabia	0.80 (0.80)	0.64	0.83[a]
Abu Dhabi, U. A. Em.	0.80 (0.75)	0.81	0.75[a]
Dubai, U. A. Em.	0.83 (0.75)	0.75	
Damascus, Syria	0.83 (0.78)	0.93	
Tripoli, Libya	0.83 (0.89)	0.89	
Cairo, Egypt	0.84 (0.77)	0.71	0.90[a]

Table S3: Mean normalised NO_2 weekly profiles averaged over the studied cities in different countries of the world.

Country	M	T	W	T	F	S	S
Arab Emirates	1.01	1.06	1.08	1.04	0.86	0.92	1.04
Saudi Arabia	1.02	1.07	1.05	1.06	0.79	1.00	1.01
Syria	1.02	1.06	1.04	1.04	0.87	1.01	0.95
Iraq	1.01	1.01	1.07	1.01	0.94	0.99	0.98
Iran	1.03	1.05	1.06	0.94	0.90	1.02	1.00
Israel	1.13	1.01	1.07	1.13	0.87	0.77	1.02
Germany	1.03	1.04	1.09	1.09	1.08	0.93	0.73
Spain	1.02	1.09	1.13	1.10	1.15	0.84	0.68
Great Britain	1.14	1.13	1.11	1.06	1.20	0.69	0.66
Poland	1.01	1.03	1.10	1.04	1.07	0.90	0.85
Greece	1.06	1.03	1.06	1.05	1.12	0.87	0.80
Italy	1.01	1.08	1.07	1.11	1.08	0.89	0.77
Ukraine	1.00	1.02	1.01	1.05	1.02	0.98	0.92
Turkey	0.98	1.00	1.07	1.05	0.98	1.02	0.90
Japan	1.01	1.08	1.18	1.05	1.03	0.94	0.70
Korea	1.04	1.11	1.11	0.99	1.00	0.91	0.84
China	1.03	1.01	0.98	0.99	0.97	1.06	0.97
Taiwan	0.96	1.04	1.06	1.01	1.07	1.01	0.86
Philippines	0.98	0.99	1.04	1.02	0.93	1.06	0.97
Malaysia	1.00	1.04	1.06	1.00	0.95	1.02	0.91
Vietnam	1.02	0.96	1.02	1.00	1.05	1.03	0.92
Indonesia	1.07	1.00	1.06	1.00	0.99	0.97	0.91
India	1.00	1.00	1.04	0.98	1.01	1.03	0.94
Bangladesh	1.05	1.00	0.98	1.00	1.00	1.01	0.97
Canada	1.10	1.06	1.09	1.02	1.07	0.84	0.81
U.S.	1.01	1.06	1.09	1.09	1.09	0.89	0.77
Argentina	0.93	1.05	1.07	1.02	1.16	0.99	0.77
Venezuela	1.00	1.00	1.05	1.02	1.02	0.99	0.92
Australia	1.03	1.10	1.09	1.11	1.06	0.84	0.77
Morocco	1.03	1.00	1.04	1.01	0.98	1.03	0.91
Guinea	0.98	1.04	1.09	0.96	1.08	0.95	0.90
Nigeria	1.01	1.00	1.00	1.03	0.99	1.01	0.97
South Africa	1.05	1.05	1.04	1.01	1.09	0.93	0.82

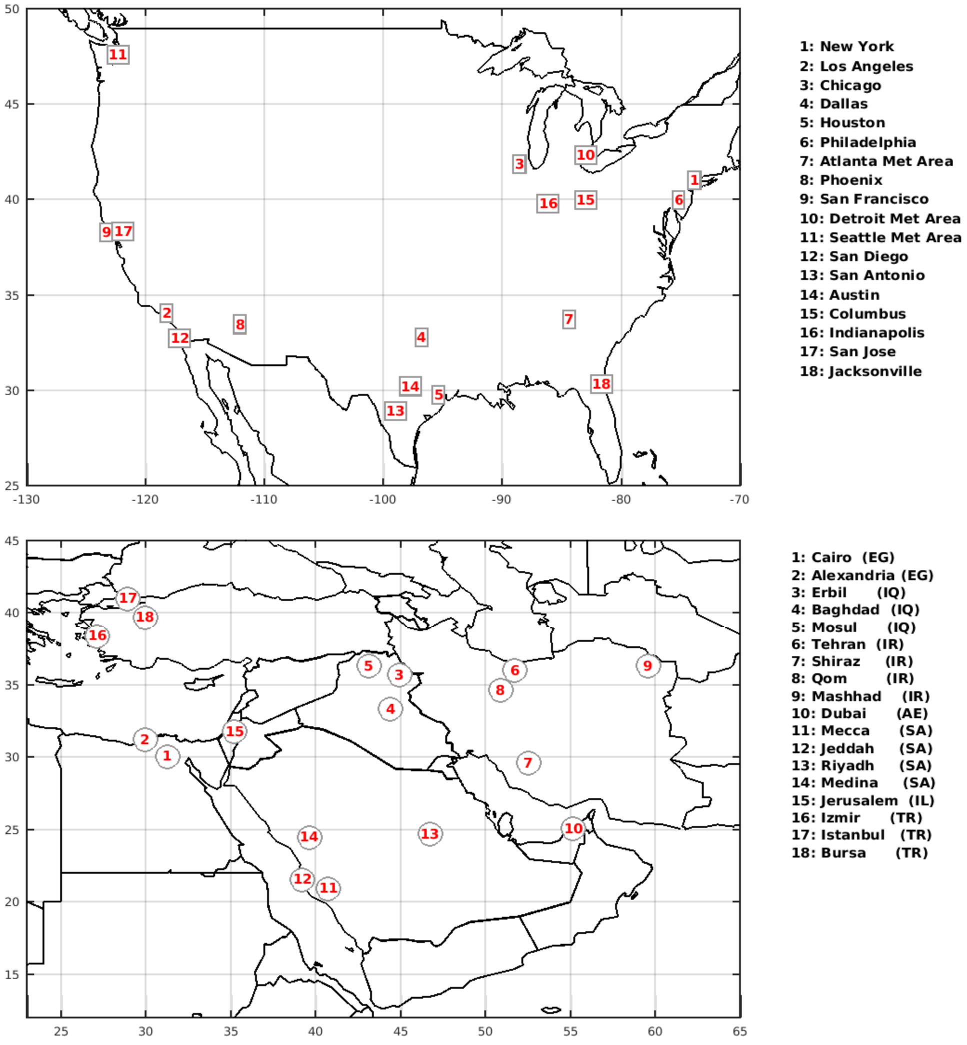

Figure S1: Location of the cities in the U.S. and the Middle East shown in Figures 4 and 6 of the main article. The administrative units are from the 2016 EUROSTAT countries dataset available in 60m resolution at https://ec.europa.eu/eurostat/web/gisco/geodata/reference-data/administrative-units-statistical-units/countries. The map is generated using MATLAB R2013a (8.1.0.604), Natick, Massachussetts, The MathWorks Inc.

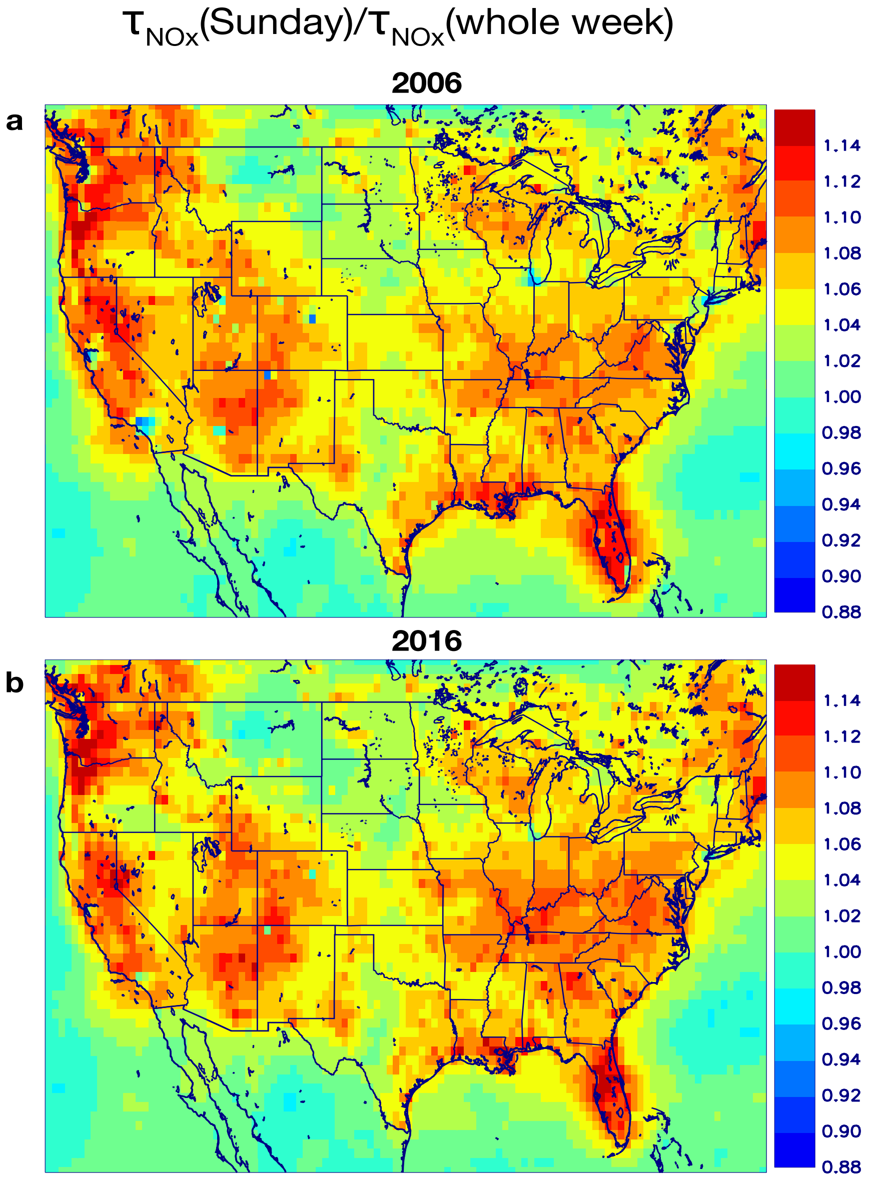

Figure S2: Ratio of the yearly averaged NOx lifetime on Sunday to the yearly averaged NOx lifetime for the whole week (a) in 2006 and (b) in 2016, as calculated by the MAGRITTE model. The same meteorological fields and natural emissions are used in both simulations, which differ only by their anthropogenic emissions.
